# The impact of life stressors on depression in college students: the chain mediating role of environmental adaptation and coping styles

**DOI:** 10.3389/fpsyg.2025.1558407

**Published:** 2025-06-24

**Authors:** Huanhuan Yang, Jing Zhao, Yuan Qin, Yongning Qian

**Affiliations:** ^1^Mental Health Education Center, Shaanxi Technical College of Finance & Economics, Xianyang, China; ^2^School of Accounting, Shaanxi Technical College of Finance & Economics, Xianyang, China; ^3^School of Humanities and Arts, Shaanxi Technical College of Finance & Economics, Xianyang, China

**Keywords:** life stressors, depression, environmental adaptation, coping styles, college students

## Abstract

**Objective:**

To explore the chain mediating role of environmental adaptation and coping styles between life stressors and depression among university students.

**Methods:**

Twelve thousand, one hundred ninety-eight college students were investigated with Student Life Stress Inventory (SLSI), Depressive Episode Scale (DES), Environmental Adaptation Scale (EAS), and Coping Style Scale (CSS). The chain intermediary model was constructed and verified with SPSS.

**Results:**

Life stressors and coping styles were positive predictors of depression, while environmental adaptation was a negative predictors. The direct impact of life stressors on depression was significant. Environmental adaptation and coping styles exhibited both independent mediating effects and a chain mediating effect.

**Conclusion:**

Life stressors can directly lead to depression, and indirectly exacerbate depression through environmental adaptation and coping styles. Therefore, reducing burden, enhancing environmental adaptability and learning appropriate coping strategies can effectively prevent depression.

## 1 Introduction

Depression is a widespread mood disorder characterized by low mood, lack of vitality, and reduced speech and motor activity (Smith, 2014). The detection rate of depression among Chinese college students is about 24.71%, and the incidence rate is increasing continuously. Depression can disrupt the emotional equilibrium and academic performance of college students. Moreover, it may also lead to more extensive social functional impairments and long-term psychological issues. Currently depression has emerged as a global health issue. Therefore investigating the factors and mechanisms influencing depression is crucial for the development of effective intervention strategies.

Previous research indicates that depression is influenced by various factors, with life stressors being a well-recognized direct and significant trigger (Wang, 2022). Life stressors are events or situations encountered daily that challenge and pressure an individual’s psychological state (Moon et al., 2024). It widely encompasses environmental, social, psychological, and physiological aspects. Research has revealed a significant positive correlation between life stressors and depression, particularly the accumulation of stressful life events, which significantly increases the risk of depression (Chang et al., 2016; Acharya et al., 2018). Stressors of college students have a significant positive predictive effect on the changes in their depressive levels. Specifically, an increase in life stressors directly leads to an exacerbation of depressive symptoms (Yu et al., 2014). This result may be attributed to a series of complex emotional, physiological, and behavioral responses by life stressors (Liu et al., 2023).

Individual’s environmental adaptability as a psychological trait has been proven to regulate the relationship between objective environment and depression. That is, individuals with relatively weak environmental adaptability may show higher sensitivity when facing life stressors, thereby increasing the risk of depression (Dong and Chao, 2025). The study indicates a strong association between insufficient adaptability and depression. Individuals with inadequate adaptability are more likely to face greater challenges when confronted with life stressors, which in turn may worsen their depressive symptoms (Zhou et al., 2015). In addition, the research also reveals that improving an individual’s adaptability has a certain alleviating effect on students’ depression (Song and Li, 2020).

Much research has verified the mediating role of coping styles between life stressors and depression. Not only can coping styles independently constitute a key bridge. In college students, it can also construct a chain mediation effects with neuroticism or psychological capital (Shuangjin et al., 2018). The choice of coping styles shows a high degree of individual differences and adjusts with the dynamic changes of environmental conditions.

Environmental adaptation and coping styles exhibit a series of complex, sequential mediating effects. Individuals with poorer environmental adaptability have a greater tendency to adopt negative coping styles, such as self-blame, alienation, and self-isolation (Qi L et al., 2018). The findings indicate that environmental adaptation can influence depression through different coping styles. Positive coping styles are negatively correlated with depression, indicating that they can alleviate depression. On the contrary, there is a positive correlation, meaning the exacerbating of depression (Yao et al., 2019).

Although existing research has explored the relationship between life stressors, environmental adaptability, and coping styles with depression, there is a lack of research on their possible interactions and serial mediating effects. In the context of increasing pressure on college students as society transforms, this study focuses on whether life stressors can positively predict depression in college students and how environmental adaption and coping styles affect the depression through serial mediating effects. By studying these internal mechanisms, it will provide a new perspective for understanding the psychological pathways of depression and provide theoretical support for the development of targeted intervention strategies.

Based on literature review and theoretical analysis, we propose the following hypotheses:

*H1*: Life stressors will positively predict depression in college students.

*H2*: Coping styles will mediate the relationship between life stressors and depression.

*H3*: Environmental adaptation will mediate the relationship between life stressors and depression.

*H4*: Coping styles and Environmental adaptation will function as chain mediators in the relationship between life stressors and depression.

The sequential mediation model was as follows: Life stressors → Environmental adaptation → Coping styles → depression.

## 2 Methods

### 2.1 Subjects and data collection

Utilizing convenience sampling, this study selected 12,198 students from Shaanxi University of Finance and Economics in June 2023. Prior to the survey, the content and purpose of the investigation were explained, and informed consent was obtained from the college students. The questionnaires were distributed by trained investigators using “Wenjuanxing” which is an online survey platform. And the students were assembled in the classroom to fill out the questionnaire. To control for potential common method bias, measures were implemented by anonymity and lie detection. The research subjects include students from different grades, majors, and family backgrounds. Ultimately, 12,198 valid responses were retained after excluding invalid ones. The sample consisted of 3,950 males and 8,248 females, with an average age of 19.86 years.

### 2.2 Measures

#### 2.2.1 Life stressors

The study employed the “Student-Life Stress Inventory (SLSI)” developed by Gadzella. This scale was translated and revised by Wang Xin, and simplified by Zheng Linke. It consists of 4 dimensions: frustration stimulation, life changes, internal and external pressures, self-imposed, using the 5-point rating scale (“1 ~ 5” representing “none to always”). In this study, it has good reliability and validity (Cronbach’s Alpha is 0.837, KMO test value is 0.912).

#### 2.2.2 Depression

The study utilized the “Depression Onset Scale” revised by Zheng Linke based on the 10 symptoms of depression onset in CCMD-3. This scale includes 9 dimensions, with a 5-point rating scale (“1 ~ 5” representing “none to always”). In this study, it has good reliability and validity (Cronbach’s Alpha is 0.782, KMO test value is 0.870).

#### 2.2.3 Environmental adaptation

The study employed the “Environmental Adaptation Scale” designed by Zheng Linke. This scale consists of 9 items, with a scoring standard of 1 = present, 0 = absent. In this study, it has a good reliability and validity in this study (Cronbach’s Alpha is 0.678, KMO test value is 0.736).

#### 2.2.4 Coping styles

The study utilized the “Coping Strategies Scale” revised by Xiao Jihua. This scale includes 6 dimensions: internalization, seeking help, self-blame, self-help, fantasy, withdrawal, excuse, lying flat, none, with a scoring standard of 1 = present, 0 = absent. In this study, it has a good information degree in this study (Cronbach’s Alpha is 0.646, KMO test value is 0.763).

### 2.3 Statistical analysis

This study used SPSS 27.0 and Amos 23.0 for data analysis. Firstly, descriptive statistics (mean, standard deviation) were calculated for all variables and the correlation between life stressors, environmental adaptation, coping styles and Depression was explored. The reliability of the scales was assessed using Cronbach’s alpha. Correlation analysis was conducted using Spearman’s correlation test. The SPSS macro PROCESS was used to test the mediating role. The fit of the model was evaluated using AMOS.

## 3 Results

### 3.1 Common method bias test

The Harman single-factor test was used for an unrotated exploratory factor analysis of all 44 items of the variables. The results show that there were 10 factors with eigenvalues greater than 1, and the first factor explained 19.7% of the variance, which was below the critical standard of 40%, indicating that there was no obvious common method bias in this study.

### 3.2 Descriptive statistics and correlation analysis

As shown in Table 1, there are significant correlations between life stressors, environmental adaptation, coping styles and depression (*p* < 0.01), indicating that it is suitable for further mediation analysis.

**Table 1:** Mean, standard deviation, and Pearson’s correlation of all variables (*n* = 12,198).

Variables	M ± SD	1	2	3	4
1 Depression	0.115 ± 0.190	–			
2 Life stressors	0.190 ± 0.209	0.573^**^	–		
3 Environmental adaptation	0.887 ± 0.165	−0.499^**^	−0.347^**^	–	
4 Coping styles	0.170 ± 0.193	0.477^**^	0.506^**^	−0.278^**^	–

*p < 0.05, **P < 0.01, *** < 0.001, M, mean; SD, standard deviation.

### 3.3 Structural equation construction and mediation effect test

The SPSS macro PROCESS 4.2 was used, and Model 6 was selected for multiple hierarchical regression analysis. Life stressors were used as the independent variable, and depression as the dependent variable, to test the mediating role between environmental adaptation and coping styles. The results are shown in Table 2. All paths reached a statistically significant level: life stressors can negatively predict environmental adaptation (*β* = −0.39, *t* = −63.53, *p* < 0.001), and can directly positively predict coping styles (*β* = 0.42, *t* = 49.20, *p* < 0.001); environmental adaptation can negatively predict coping styles (*β* = −0.06, *t* = −5.79, *p* < 0.001); life stressors can positively predict depression (*β* = 0.36, *t* = 45.01, *p* < 0.001), and environmental adaptation can negatively predict depression (*β* = −0.08, *t* = −8.12, *p* < 0.001), coping styles can positively predict depression (*β* = 0.29, *t* = 37.3, *p* < 0.001). Then Hypothesis 1 was supported.

**Table 2:** Regression analysis of life stressors, depression, environmental adaptation, and coping styles (*n* = 12,198).

Variable	Environmental adaptation			Coping styles			Depression		
	*β*	SE	*t*	*β*	SE	*t*	*β*	SE	*t*
Constant	0.96	0.00	548.57	0.15	0.01	13.88	0.06	0.01	6.90
Life stressors	−0.39	0.01	−63.53^***^	0.42	0.01	49.20^***^	0.36	0.01	45.01^***^
Environmental adaptation				−0.06	0.01	−5.79^***^	−0.08	0.01	−8.12^***^
Coping styles							0.29	0.01	37.3^***^
*R* ^2^	0.25			0.23			0.40		
*F*	4035.60^**^			1822.14^**^			2728.79^**^		

*P < 0.05, **P < 0.01, *** < 0.001.

**Figure 1:**
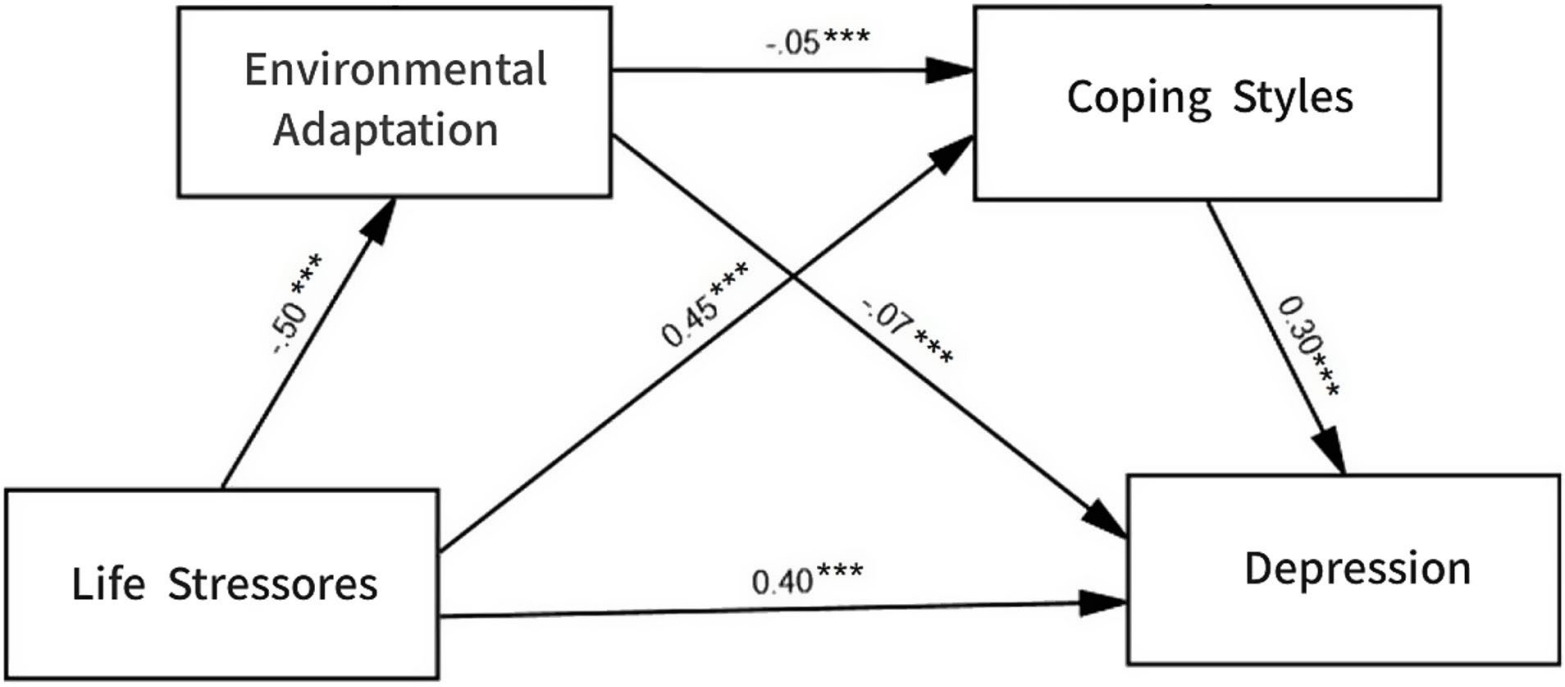
Path diagram of the impact of life stressors on depression.

The Bootstrap method was used to test the mediating effect, with 5,000 resamplings to calculate the 95% confidence interval. If the confidence interval does not contain the value of 0, it indicates statistical significance. As shown in Table 3, the results indicated that the total indirect effect (0.16) of environmental adaptation and coping styles has a Bootstrap 95% confidence interval that does not contain the value of 0, indicating that the two mediating variables have a significant mediating effect between life stressors and depression. It was composed of three indirect effects: (1) the indirect effect produced by life stressors → environmental adaptation → depression (0.03); (2) the indirect effect produced by life stressors → coping styles → depression (0.13); (3) the indirect effect produced by life stressors → environmental adaptation → coping styles → depression (0.01). The confidence intervals of the three indirect effects do not contain the value of 0, indicating that the indirect effects produced by the three paths all reached a significant level. Based on the above analysis, the model and standardized path coefficients were presented in Figure 1. Therefore, Hypotheses 2, 3, and 4 are supported.

**Table 3:** Mediation effect of environmental adaptation and coping styles between life stressors and depression.

Path	Indirect effect value	Boot std. error	Boot CL Lower	Boot CI Upper
Total indirect effect	0.16	0.01	0.14	0.17
Life stressors → Environmental adaptation → Depression	0.03	0.01	0.02	0.04
Life stressors → Coping styles → Depression	0.12	0.01	0.11	0.13
Life stressors → Environmental adaptation → Coping styles → Depression	0.01	0.00	0.00	0.01

## 4 Discussion

### 4.1 Life stressors lead to depression among college students

This study found that life stressors can directly and positively predict depression (*β* = 0.36, *t* = 45.01, *p* < 0.001), which is consistent with previous research findings (Yuanzhi et al., 2024; Rivera et al., 2017). In recent years, college students have been increasingly confronted with a multitude of life stressors, including academic pressures, interpersonal challenges, financial constraints, and intense social competition. These interconnected stressors have emerged as significant contributors to the rising prevalence of depression among this demographic, adversely affecting their academic performance and overall quality of life. Consequently, students experiencing depression often exhibit academic decline, strained interpersonal relationships, and, in severe cases, engage in extreme behaviors (Lardier et al., 2020). Despite the growing societal concern over this issue and the enhanced focus on mental health education within universities, the effectiveness of current interventions remains suboptimal.

To address this pressing issue, it is imperative for higher education institutions to incorporate comprehensive stress management modules into their mental health curricula, thereby equipping students with evidence-based strategies for stress alleviation. Furthermore, universities should implement therapeutic interventions such as art therapy and structured group counseling sessions to facilitate stress reduction. Additionally, a tripartite collaborative framework involving families, educational institutions, and community organizations should be established to foster a supportive and less stressful social environment for college students.

### 4.2 Environmental adaptation plays a crucial role in alleviating depression

This study found that environmental adaptation negatively predicts depression, meaning that the stronger the capacity for environmental adaptation, the lower the risk of depression. The findings are consistent with previous research results. Researchers have analyzed the relationship between environmental adaptation and depression from multiple perspectives. Good environmental adaptation is a protective factor against depression. College students with lower environmental adaptation abilities, such as emotional adaptation, self-adaptation, and campus adaptation, lead to lower self-evaluation and thus depression (Zhou et al., 2015). The specific genetic polymorphisms (such as the 5-HTT gene) can affect an individual’s response to environmental stress, thereby influencing depression (Caspi et al., 2003).

The study found that environmental adaptation plays a mediating role between life stressors and depression. Research indicates that individuals with good environmental adaptation are less affected by life stressors (Zhang et al., 2014). The reasons are that, firstly, a higher adaptation can enhance an individual’s perception and utilization of environmental resources, thereby reducing the accumulation of negative emotions (Lehrer et al., 2017); secondly, individuals with higher environmental adaptation can better regulate emotional responses, thus better coping with life’s pressures and challenges (Xu, 2019; Wong and C, 2015). Therefore, environmental adaptation is an important protective factor that can reduce the likelihood of depression. However, this effect does not exist in isolation; it interacts with other factors such as social support, family environment, and personal psychological traits, collectively affecting an individual’s mental health. Therefore, in preventing and treating depression, these factors need to be considered comprehensively, and diversified intervention measures should be adopted.

### 4.3 Active coping styles reduce depression

This study found that coping styles act as a mediator between life stressors and depression, a finding consistent with previous research results. Active coping styles potentially help individuals handle stress more effectively and reduce depressive emotions; while passive coping styles may lead to persistent problems and an increase in the accumulation of depressive emotions (Guo and Ma, 2022; Liu, M. et al., 2024).

Environmental adaptation indirectly affects depression by influencing coping styles. Individuals with higher environmental adaptation are more capable of effectively using cognitive regulation, emotional management, and behavioral efforts to handle problems and conflicts when facing stress (Jia, 2012; Xu et al, 2023). Therefore, good environmental adaptation may promote the development of more mature coping styles in individuals, thereby reducing the accumulation of depressive emotions. However, some studies have pointed out that although there are gender differences in the relationship between active coping strategies and depression scores, gender plays a moderating role in the first half of this mediation model, implying that the impact of coping strategies on depression may be influenced by individual characteristics (Xuexiao et al., 2022). In addition, family environment and dormitory interpersonal relationships are significantly negatively correlated with passive strategies and depression, while family environment, active strategies, and dormitory interpersonal relationships are significantly positively correlated with each other, indicating that family and social environmental factors also have an important impact on individuals’ coping strategies and depressive emotions (Lee and Park, 2020).

At the same time, individual factors such as personality traits, social support, and psychological capital also affect individuals’ coping styles and depressive emotions (Shuangjin et al., 2018). Therefore, mental health interventions for the college student population should consider these factors to promote the development and application of more effective coping strategies.

### 5 Research significance and limitations

This study, employing a quantitative research method, explores the impact of life stressors on depression and the chain mediating effects of environmental adaptation and coping styles, which holds theoretical and practical significance. Theoretically, this study reveals the complex relationships among life stressors, environmental adaptation, coping styles, and depression, thereby providing a theoretical foundation for subsequent studies. Additionally, by the exploration of chain mediation effects, it enhances the understanding of the mechanisms underlying depression, particularly the internal mechanisms through which environmental adaptation and coping styles jointly influence depression. Additionally, the quantitative research methods employed in this study serve as a reference for analogous research, contributing to the advancement of quantitative research in the field of mental health.

Practically, this study offers a new perspective for universities to prevent and intervene in depression among students. First, studies have found that life stressors directly lead to depression and indirectly exacerbate it through environmental adaptation and coping styles. Therefore, universities can effectively prevent depression by reducing students’ academic burdens, enhancing their environmental adaptation capabilities, and teaching appropriate coping strategies. For instance, universities can optimize course settings and teaching methods to reduce students’ academic pressure. Mental health educators can offer adaptation courses for freshmen to help them quickly adapt to university life, build positive interpersonal relationships, and find a suitable learning pace. Classes can also organize lectures, workshops, and group counseling to help students develop a positive mindset, learn time management, and master effective coping strategies.

This study has several limitations. First, this study employs a cross-sectional survey design, which cannot accurately infer causal relationships between variables. Future research could address this limitation by adopting longitudinal follow-up or experimental intervention designs to explore the causal relationships between life stressors, environmental adaptation, coping styles, and depression. Second, this study focuses on students from a single university, which may limit the generalizability of the findings. Future research should consider students from different types of institutions to validate the broader applicability of these findings. Third, this study only examines the mediating effects of environmental adaptation and coping styles, and there may be other mediating variables that have not been included. For example, social support, personality traits, and family background may also mediate the relationship between life stressors and depression. Future research should further explore these potential mediating variables to gain a more comprehensive understanding of the mechanisms underlying depression.

## Data Availability

The raw data supporting the conclusions of this article will be made available by the authors, without undue reservation.
